# Predicting axillary lymph node metastasis in breast cancer using the similarity of quantitative dual-energy CT parameters between the primary lesion and axillary lymph node

**DOI:** 10.1007/s11604-022-01316-8

**Published:** 2022-07-25

**Authors:** Kanako Terada, Hiroko Kawashima, Norihide Yoneda, Fumihito Toshima, Miki Hirata, Satoshi Kobayashi, Toshifumi Gabata

**Affiliations:** 1 Department of Radiology, Kanazawa University Graduate School of Medical Sciences, 13-1 Takaramachi, Kanazawa, Ishikawa, 920-8640 Japan; 2 Department of Quantum Medical Imaging, Kanazawa University Graduate School of Medical Sciences, 13-1 Takaramachi, Kanazawa, Ishikawa, 920-8640 Japan; 3 Department of Breast Oncology, Kanazawa University Graduate School of Medical Sciences, 13-1 Takaramachi, Kanazawa, Ishikawa, 920-8640 Japan

**Keywords:** Breast cancer, Lymph node metastasis, Dual-energy CT

## Abstract

**Purpose:**

To evaluate the similarity of quantitative dual-energy computed tomography (DECT) parameters between the primary breast cancer lesion and axillary lymph node (LN) for predicting LN metastasis.

**Materials and methods:**

This retrospective study included patients with breast cancer who underwent contrast-enhanced DECT between July 2019 and April 2021. Relationships between LN metastasis and simple DECT parameters, similarity of DECT parameters, and pathological and morphological features were analyzed. ROC curve analysis was used to evaluate diagnostic ability.

**Results:**

Overall, 137 LNs (39 metastases and 98 non-metastases) were evaluated. Significant differences were observed in some pathological (nuclear grade, estrogen receptor status, and Ki67 index) and morphological characteristics (shortest and longest diameters of the LN, longest-to-shortest diameter ratio, and hilum), most simple DECT parameters, and all DECT similarity parameters between the LN metastasis and non-metastasis groups (all, *P* < 0.001–0.004). The shortest diameter of the LN (odds ratio 2.22; 95% confidence interval 1.47, 3.35; *P* < 0.001) and the similarity parameter of 40-keV attenuation (odds ratio, 2.00; 95% confidence interval 1.13, 3.53; *P* = 0.017) were independently associated with LN metastasis compared to simple DECT parameters of 40-keV attenuation (odds ratio 1.01; 95% confidence interval 0.99, 1.03; *P* =0.35). The AUC value of the similarity parameters for predicting metastatic LN was 0.78–0.81, even in cohorts with small LNs (shortest diameter < 5 mm) (AUC value 0.73–0.78).

**Conclusion:**

The similarity of the delayed-phase DECT parameters could be a more useful tool for predicting LN metastasis than simple DECT parameters in breast cancer, regardless of LN size.

## Introduction

Globally, breast cancer is the most commonly diagnosed cancer and the leading cause of cancer-related deaths in women [1]. The morbidity and mortality associated with breast cancer are increasing. Before breast cancer treatment, ultrasonography (US), mammography, magnetic resonance imaging (MRI), positron emission tomography (PET), and computed tomography (CT) are performed to determine the stage of the cancer, spread of the lesion, and method of treatment. The presence of metastatic axillary lymph nodes (LNs) affects the staging, operative method, and prognosis [2–4]. Therefore, non-invasive imaging is clinically valuable for improving the diagnostic ability of LN metastasis.

Dual-energy CT (DECT) is a technology that collects data from two different energy spectra. Therefore, the difference in attenuation at the two energies can be used to differentiate and quantify material decomposition [5]. Based on this technology, virtual monochromatic spectral images, material density images, and virtual pre-contrast images can be created. DECT improves the contrast of lesions and improves visibility in breast, gastrointestinal stromal, and lung cancers [6–8]. Additionally, DECT can obtain various quantitative values and contribute to the evaluation of lesions. Recently, several studies have shown that quantitative parameters from DECT can be used to evaluate the diagnosis or characterization of lesions in various tumors [9]. To date, studies have been performed to differentiate benign and malignant adrenal glands, lungs, ovaries, kidneys, or liver lesions [10–14] and to predict tumor histology of the breast, rectum, or parotid glands [15–17].

In previous studies of DECT for breast cancer, Zhang et al. reported the diagnostic performance of preoperative metastatic sentinel LNs (SLNs), and the accuracy of the venous phase slope of the spectral Hounsfield unit curve (λHU), one of the quantitative DECT parameters that represents the slope of the CT value between 40 and 70 keV for detecting metastatic SLNs, was 90.5%; moreover, the venous-phase λHU had a higher specificity and accuracy than the morphologic parameters (*P* < 0.001) [18]. Volterrani et al. investigated DECT for primary breast cancer to differentiate tumor histotypes and reported receiver operating characteristic (ROC) curves derived from lesion iodine concentration (IC) (one of the quantitative DECT parameters); they showed that the optimal thresholds for distinguishing infiltrating carcinomas (invasive ductal carcinoma [IDC] and invasive lobular carcinoma [ILC]) from other lesions was 1.70 mg/mL (sensitivity, 94.9%; specificity, 93.0%; area under the curve [AUC], 0.968) [19]. In earlier reports, primary lesions and LN metastasis were evaluated independently, and no study has evaluated the relationship between the primary and metastatic lesions.

DECT parameters can be used to predict LN metastasis or tumor histology in breast cancer [18–20]. However, these DECT parameters may be potentially influenced by differences in the CT scanner, scanning protocols, and contrast injection protocols. In this study, we aimed to evaluate the similarity of quantitative DECT parameters between the primary lesion and axillary LN to predict the presence of LN metastasis in patients with breast cancer. We hypothesized that the primary lesion and LN metastasis would have some similarity in the DECT parameters and that assessing the similarity could be useful in diagnosing LN metastasis i.e., a CT scanning factor-independent assessment method.

## Materials and methods

### Ethical considerations

The research ethics committee of the Graduate School of Medical Sciences approved the study protocol (approval number: 2020-253). All study procedures were performed in accordance with the ethical standards of the responsible committee on human experimentation (institutional and national), as determined by the Helsinki Declaration. The requirement for informed consent was waived owing to the retrospective study design.

### Patients

**Fig. 1:**
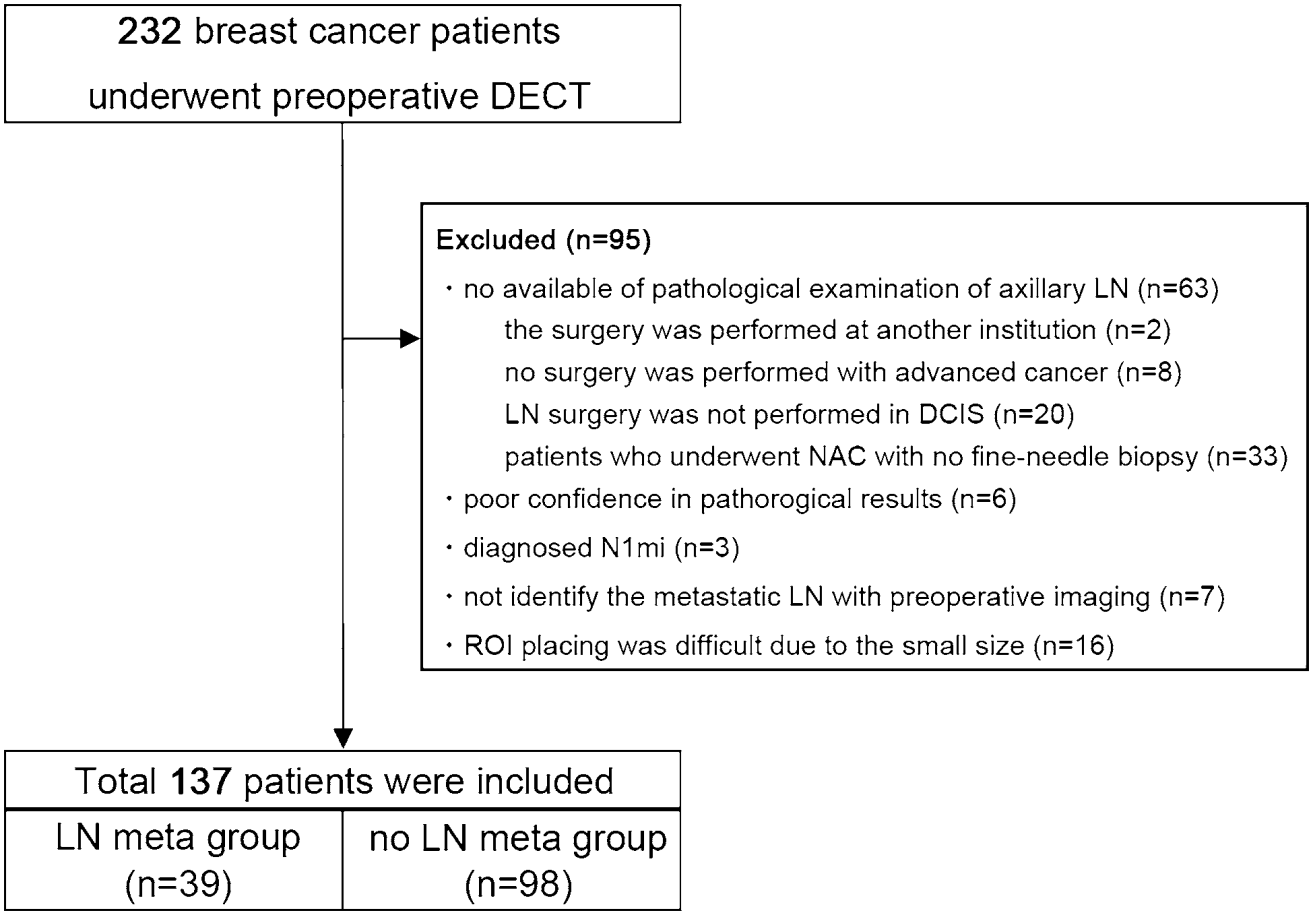
Flow chart of the study sample selection. *DECT* dual-energy computed tomography, *NAC* neoadjuvant chemotherapy, *LN* lymph node, *DCIS* ductal carcinoma in situ, *FNA* fine-needle aspiration cytology, *ROI* region of interest

In total, 232 consecutive patients with newly diagnosed breast cancer underwent preoperative DECT from July 2019 to March 2021 (Fig. 1). In our institution, all patients with breast cancer undergo preoperative DECT. All patients underwent needle biopsy for breast cancer diagnosis. This included 61 patients who underwent neoadjuvant chemotherapy (NAC) preoperatively, and all of them underwent DECT before NAC. We excluded patients who had no available pathologic examination of LNs (*n* = 63) because surgery was performed at another institution (*n* = 2), no surgery was performed for advanced cancer (*n* = 8), or LN surgery was not performed in ductal carcinoma in situ (DCIS) (*n* = 20); patients who underwent NAC with no fine-needle aspiration cytology (FNA) of axillary LNs (*n* = 33); patients with unreliable pathological results (*n* = 6); patients diagnosed with N1mi (*n* = 3); patients who had LN metastasis pathologically that could not be identified by normal preoperative examination imaging (*n* = 7) and patients with axillary LNs or breast primary lesions that were too small or unclear to place regions of interest (ROIs) (*n* = 16).

### Imaging

All patients underwent non-enhanced and enhanced chest-abdominal CT on a 256 multi-detector row CT scanner (Revolution CT; GE Healthcare, Chicago, IL, USA) using the fast-kilovoltage-switching dual-energy CT imaging scan mode with fast tube voltage switching between 80 and 140 kVp. Non-enhanced chest-abdominal DECT was acquired first. After the non-contrast CT scan, the non-ionic contrast medium was injected at a patient weight-dependent dose of 600 mg iodine/kg in 1 min via the antecubital vein. At 120 s after the start of the contrast injection, delayed-phase contrast-enhanced DECT was performed. The other CT scanning parameters were as follows: noise index, 10 at 5-mm thickness (GSI Assist; GE Healthcare); iterative reconstruction, advanced statistical iterative reconstruction (ASiR V; GE Healthcare) of 30%; slice thickness, 1.25 mm; slice interval, 1.25 mm; and helical pitch, 0.508:1.

### Image analysis

DECT data were analyzed using a gemstone spectral imaging (GSI) viewer on a workstation (AW server 3.2; GE Healthcare). Two radiologists (K.T. and H.K. with 9 and 31 years of experience in breast radiology, respectively) performed the measurements of primary breast cancer lesions and axillary LNs. Two radiologists placed circular ROIs on the solid areas (avoiding necrotic and cystic portions) of the tumor and LN at the slice with the largest size of the lesion. The longest and shortest diameters, hilum of the LN, and longest diameter of the primary lesion were also evaluated.

**Fig. 2:**
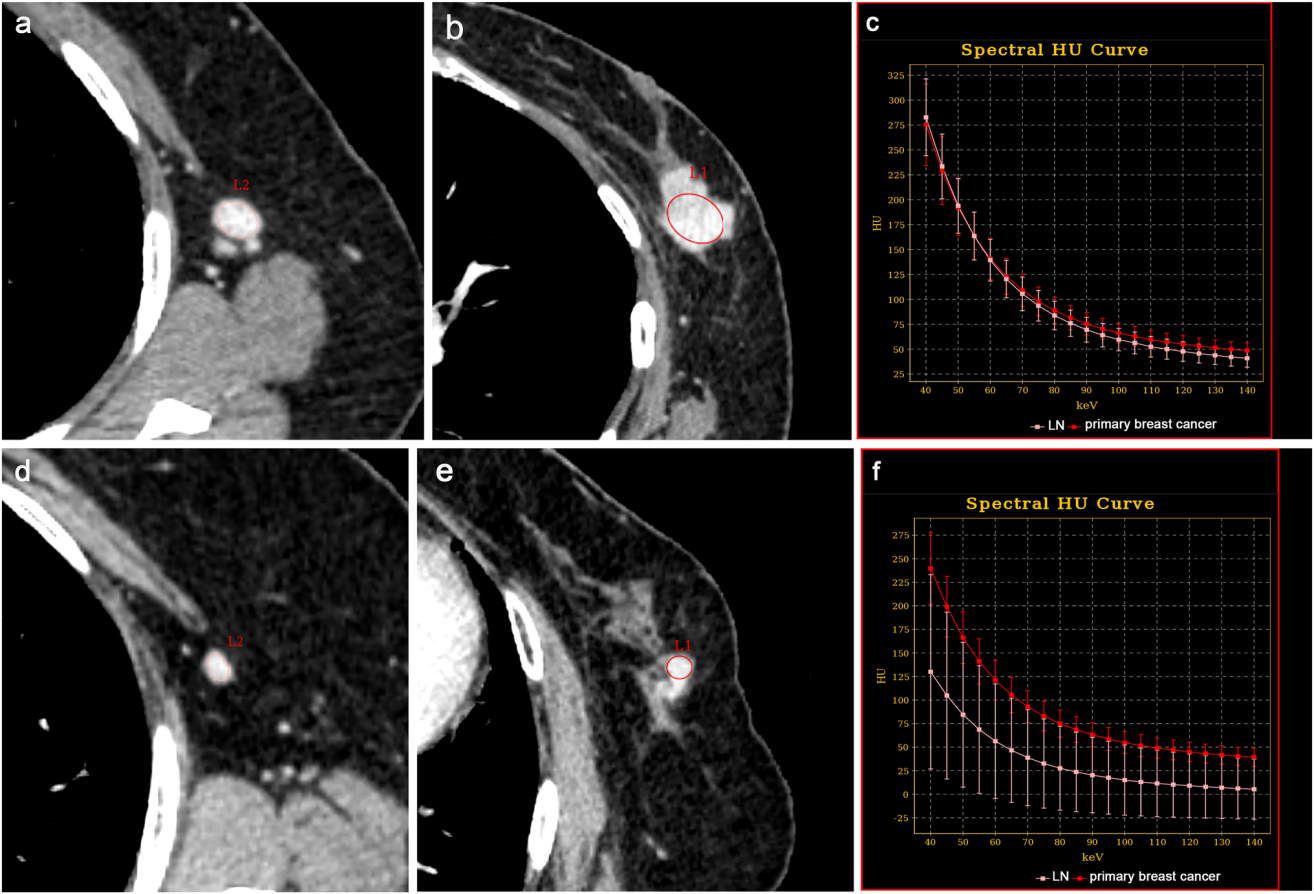
Example DECT images in the delayed phase of a 70-year-old woman’s left breast cancer with axillary LN metastasis (**a**–**c**) and an 80-year-old woman’s left breast cancer without axillary LN metastasis (**d**–**f**). **a** Contrast-enhanced monochromatic image at 40 keV of the metastatic axillary LN. The shortest diameter of the LN is 9 mm. The mean attenuation values at 40 keV and 70 keV, IC, WC, and Eff-Z are 288 HU, 108 HU, 31.8 mg/cm^3^, 1039 mg/cm^3^ and 9.34, respectively. **b** Contrast-enhanced monochromatic image at 40 keV for primary breast cancer. The mean attenuation values at 40 keV and 70 keV, IC, WC, and Eff-Z are 284, 112 HU, 30.6 mg/cm^3^, 1030 mg/cm^3^ and 9.28, respectively. **c** The graph shows the spectral HU curve of LN metastasis and primary breast cancer. The λHU of LN metastasis and breast cancer are 6.0 HU/keV and 5.7 HU/keV, respectively. The RODs of DECT parameters between the primary lesion and axillary LN in this case are 0.014 for attenuation at 40 keV, 0.036 for attenuation at 70 keV, 0.053 for the λHU, 0.039 for the IC, 0.0087 for the WC and 0.0086 for the Eff-Z. **d** Contrast-enhanced monochromatic image at 40 keV with no metastatic axillary LN. The shortest diameter of the LN is 5 mm. The mean attenuation values at 40 keV and 70 keV, IC, WC, and Eff-Z are 130 HU, 39 HU, 16.2 mg/cm^3^, 1021 mg/cm^3^, and 8.55, respectively. **e** Contrast-enhanced monochromatic image at 40 keV for primary breast cancer. The mean attenuation values at 40 keV and 70 keV, IC, WC, and Eff-Z are 240 HU, 93 HU, 26 mg/cm^3^, 1032 mg/cm^3^ and 9.09, respectively. **f** The graph shows the λHU of the non-metastatic LN and the breast cancer. λHU of LN non-metastasis and primary breast cancer are 3.0 HU/keV and 4.9 HU/keV, respectively. The RODs of DECT parameters between the primary lesion and axillary LN in this case are 0.46 for attenuation at 40 keV, 0.58 for attenuation at 70 keV, 0.39 for the λHU, 0.38 for the IC, 0.01 for the WC and 0.059 for the Eff-Z. *DECT* dual-energy computed tomography, *LN* lymph node, *IC* iodine concentration, *WC* water concentration, *Eff-Z* effective atomic number, *HU* Hounsfield units, *λHU* slope of the spectral Hounsfield unit curve, *ROD* rate of difference

DECT quantitative parameters, including the attenuation at 40 and 70 keV, IC, water concentration (WC), and effective atomic number (Eff-Z), were calculated using the GSI viewer at the workstation. The attenuation at 70 keV was selected because 120-kVp scanning in conventional polychromatic images has an average energy of approximately 70 keV [21], and at low energies, attenuation at 40 keV was evaluated frequently in previous reports [18–20]. IC and WC (in milligrams per cubed centimeter) were calculated on the iodine and water images as the base material. Eff-Z is atomic number assuming that the material in one voxel is made of one element and it is obtained from the attenuation coefficient of DECT. Additionally, to minimize variations caused by individual differences in cardiac function and blood flow dynamics between patients, the IC, WC, and Eff-Z of the regions were, respectively, divided by the IC, WC, and Eff-Z of the aorta to obtain the normalized values for each parameter. The λHU was defined as the difference between the CT value at 40 keV and that at 70 keV divided by the energy difference (30 keV) $\left( {\lambda {\text{HU}} = \frac{{{\text{The mean CT attenuation at }}40{\text{keV}} - {\text{ The mean CT attenuation at }}70{\text{keV}}}}{{30{\text{ keV}}}}} \right)$. An example of DECT images and ROIs for the assessment of the quantitative measurements are shown in Fig. 2a–f.

The rate of difference (ROD) between the DECT parameters of the primary lesion and that of the LN was calculated using the following formula: $\frac{{\left| {{\text{Lymph}} {\text{node}} {\text{value}} - {\text{Primary}} {\text{lesion}} {\text{value}}} \right|}}{{{\text{Primary}} {\text{lesion}} {\text{value}}}}$ as a new index. It reflects the similarity between the primary lesion and LN of DECT parameters; the closer it is to 0, the more similar the parameters.

### Pathological evaluation

The histological diagnosis was performed by a single pathologist with 19 years of experience in breast histological evaluation. A core biopsy of the tumor and FNA of the LNs suspected of metastasis was performed for diagnosis. The nuclear grade and immunohistochemical data, including the estrogen receptor (ER), progesterone receptor (PgR), and human epidermal growth factor receptor 2 (HER2) were obtained from patients’ medical records.

### Criteria for one-to-one matching of LNs

Axillary LN dissection or SLN biopsy was performed according to the stage of the patients. SLNs were identified by using a combination of radioisotope labeling and the blue-dye method during surgery, and all patients underwent preoperative lymphoscintigraphy in combination with single photon emission CT (SPECT)/CT scan. Details of the excised LNs (location, size, and the radioactive count and staining of SLN) were recorded. We determined metastatic LNs on the DECT images in the following two ways: identified using US images of LNs that were positive for US-FNA, or identified using SPECT/CT of preoperative lymphoscintigraphy in the patients with positive SLN. If none of the excised LNs were deemed metastatic, all the corresponding LNs on the DECT images were considered non-metastatic. One LN was analyzed per patient.

### Statistical analysis

DECT parameters, pathologic characteristics, and morphologic parameters were compared between the LN metastasis and LN non-metastasis groups using the Mann–Whitney *U* test, Fisher exact test, or chi-squared test. Multivariable binary logistic regression analyses were used to determine better DECT parameters for predicting LN metastasis. A multivariate analysis with forward elimination was performed with variables that had *P *values < 0.001 in univariable analysis, and a multivariate model was constructed with pathological and morphological characteristics from lowest *P *value as the pathological and morphological model. The number of variables was considered from the number of patients, statistically. The variables that had a significant difference in the pathological and morphological model (shortest diameter of the LN) and those with the lowest *P* value among the DECT parameters (attenuation at 40 keV) and the ROD of DECT parameters (the ROD of attenuation at 40 keV) in univariate analysis were selected and used (DECT parameters with the shortest diameter of the LN model). The Spearman rank correlation coefficient was used to evaluate the correlation between primary breast cancer and the axillary LN. Since the ROD between the DECT parameters of the primary lesion and that of the LN were too small for multivariate analysis, we used a value subtracted from 1 and multiplied the value by 10 [22]. ROC curve analysis was used to evaluate the diagnostic ability of delayed-phase DECT parameters and the similarity of delayed-phase DECT parameters between the primary lesion and axillary LN. All statistical analyses were performed using SPSS (version 22; IBM Corp., Armonk, NY, YSA) or R (version 4.0.3, R Foundation for Statistical Computing, Vienna, Austria).

## Results

### Patient characteristics

Finally, 137 patients were included. The mean age of the patients was 59.7 years (range 33–87 years). There were 110 (80.3%) patients with IDC, 7 (5.1%) patients with ILC and 12 (8.8%) patients with DCIS. The study included 22 patients who underwent NAC preoperatively, and all these patients had positive results for FNA of axillary LNs. LN metastasis (LN metastasis group) was confirmed in 39 patients and 98 patients had no LN metastasis pathologically (LN non-metastasis group) (Fig. 1). The patient characteristics are summarized in Tables 1 and 2. The breast cancer size (*P* < 0.001), nuclear grade (*P* = 0.04), ER status (*P* < 0.001), and Ki67 index (*P* < 0.001) of breast cancer were higher in the LN metastasis group than in the LN non-metastasis group, and no significant difference was observed in age, histologic type, PgR status, or HER2 status between the groups. The ROI sizes were 43.0 ± 34.1 mm^2^ and 13.9 ± 19.3 mm^2^ in breast cancer lesions and axillary LNs, respectively.

**Table 1:** Pathological characteristics of the patients

Characteristics	Total	LN metastasis group	LN non-metastasis group	*P* value
	(*n* = 137)	(*n* = 39)	(*n* = 98)	
Mean age (years)	59.7 (33–87)	60.0(37–85)	59.7 (33–87)	0.98
Tumor size (mm)	22.3 (4–33)	30.7 (10–78)	19.5(4–88)	< 0.001
Histologic type				0.08
IDC	110	36	74	
ILC	7	2	5	
DCIS	12	0	12	
Others	8	1	7	
Nuclear grade				0.04
1	57	12	45	
2	17	7	10	
3	36	17	19	
NA	27	3	24	
ER status				< 0.001
Positive	105	23	82	
Negative	30	16	14	
PgR status				0.16
Positive	85	21	64	
Negative	50	18	32	
HER 2 status				0.16
Positive	16	7	9	
Negative	119	32	87	
Ki67 index (%)	29.1 (0.8–96.9)	45.5 (2.0–96.9)	21.7 (0.8–88.0)	< 0.001
≦20%	82	12	70	
> 20%	55	27	28	

*LN* lymph node, *IDC* invasive ductal carcinoma, *ILC* invasive lobular carcinoma, *DCIS* ductal carcinoma in situ, *NA* not applicable, *ER* estrogen receptor, *PgR* progesterone receptor, *HER2* human epidermal growth factor receptor 2

**Table 2:** Morphological characteristics of the axillary lymph nodes

Characteristics	Total	LN metastasis group	LN non-metastasis group	*P* value
	(*n* = 137)	(*n* = 39)	(*n* = 98)	
Shortest diameter (mm)	4.6 (2–17)	7.6 (3–17)	3.4 (2–7)	< 0.001
< 5 mm	108	12	86	
≧5 mm	29	27	12	
Longest diameter (mm)	10.9 (6–32)	14.1 (8–32)	9.7 (6–22)	< 0.001
< 10 mm	64	6	58	
≧10 mm	73	33	40	
Longest-to-shortest diameter ratio	2.9 (1.3–6.5)	2.1 (1.3–5.7)	3.2 (1.8–6.5)	< 0.001
≦2:1	45	23	22	
> 2:1	92	16	76	
Hilum				< 0.001
Present	113	15	88	
Absent	24	24	10	

*LN* lymph node

Regarding the morphologic characteristics of axillary LNs, the means of the shortest and longest diameters in all patients were 4.6 mm (range 2–17 mm) and 10.9 mm (range 6–32 mm), respectively. The LN size was larger and the ratio of the longest and shortest diameters was smaller in the LN metastasis group than in the LN non-metastasis group (both, *P* < 0.001). The number of LNs without hilum was significantly higher in the LN metastasis group than in the LN non-metastasis group (*P* < 0.001).

### Evaluation of simple DECT parameters and the similarity of DECT parameters between the groups

Significant differences were found between the two groups in each of the following DECT parameters: delayed-phase attenuation at 40 keV (*P* < 0.001), attenuation at 70 keV (*P* < 0.001), λHU (*P* < 0.001), IC (*P* < 0.001, normalized *P* < 0.001), and Eff-Z (*P* < 0.001, normalized *P* = 0.004).

There was a moderate correlation between DECT parameters (attenuation at 40 keV, attenuation at 70 keV, λHU, IC, and Eff-Z) for the axillary LN and primary lesion (Spearman correlation coefficient: 0.609–0.692, *P* < 0.05) in the LN metastasis group. However, no correlation was found in the LN non-metastasis group (Spearman correlation coefficient: 0.115–0.165, *P* = 0.105–0.258).

The ROD between the DECT parameters of the primary lesion and that of the axillary LN, which reflects the similarity between primary lesion and axillary LN, was significantly smaller in the LN metastasis group than in the LN non-metastasis group for the following parameters: delayed-phase attenuation at 40 keV and 70 keV, λHU, IC, and Eff-Z (all, *P* < 0.001) in the univariate analysis (Table 3). The lowest *P* value among the simple DECT parameters was for attenuation at 40 keV (*P* = 2.1 × 10^–8^), and that among the ROD of DECT parameters was for the ROD of attenuation at 40 keV (*P* = 2.2 × 10^–8^).

**Table 3:** Results of the univariate analysis of simple quantitative delayed-phase dual-energy computed tomography parameters and the rate of difference of those parameters between the primary lesion and axillary lymph node in both groups

Parameters	Total	LN metastasis group	LN non-metastasis group	*P* value
	(*n* = 137)	(*n* = 39)	(*n* = 98)	
Simple DECT parameters on delayed-phase				
Attenuation at 40 keV	214 ± 47	247 ± 38	204 ± 45	< 0.001
Attenuation at 70 keV	86 ± 17	99 ± 14	81 ± 16	< 0.001
λHU	4.3 ± 1.0	4.9 ± 0.9	4.0 ± 1.1	< 0.001
IC	22.8 ± 5.8	25.8 ± 4.8	21.6 ± 5.6	< 0.001
Normalized IC	0.44 ± 0.10	0.50 ± 0.10	0.42 ± 0.10	< 0.001
WC	1028 ± 0.10	1031 ± 8.4	1027 ± 12	0.04
Normalized WC	1.0 ± 0.01	1.0 ± 0.01	1.0 ± 0.01	0.28
Eff Z	9.0 ± 0.30	9.1 ± 0.23	8.9 ± 0.29	< 0.001
Normalized Eff Z	0.88 ± 0.04	0.89 ± 0.03	0.87 ± 0.04	0.004
The delayed-phase ROD of DECT parameters between the primary lesion and LN				
Attenuation at 40 keV	0.15 ± 0.17	0.05 ± 0.08	0.21 ± 0.17	< 0.001
Attenuation at 70 keV	0.18 ± 0.15	0.08 ± 0.08	0.23 ± 0.15	< 0.001
λHU	0.18 ± 0.24	0.08 ± 0.10	0.27 ± 0.26	< 0.001
IC	0.18 ± 0.24	0.08 ± 0.10	0.27 ± 0.26	< 0.001
Eff Z	0.02 ± 0.03	0.01 ± 0.01	0.04 ± 0.03	< 0.001

*DECT* dual-energy computed tomography, *λHU* slope of the spectral Hounsfield unit curve, *IC* iodine concentration, *WC* water concentration, *Eff-Z* effective atomic number, *LN* lymph node, *ROD* rate of difference

In the multivariate analysis of the pathological and morphological model, the shortest diameter of axillary LNs was most associated with LN metastasis (odds ratio [OR], 2.49; 95% confidence interval [CI]: 1.65, 3.77; *P* < 0.001). The absence of LN hilum was also associated with LN metastasis (OR, 0.21; 95% CI: 0.06, 0.72; *P* = 0.014). In the multivariate analysis that included the shortest diameter of the LN, attenuation at 40 keV, the ROD of attenuation at 40 keV (DECT parameters with the shortest diameter of the LN model), ROD of attenuation at 40 keV (OR, 2.00; 95% CI: 1.13, 3.53; *P* = 0.017) together with the shortest diameter of the axillary LN (OR, 2.22; 95% CI: 1.47, 3.35; *P* < 0.001) were independently associated with LN metastasis. Attenuation at 40 keV was not significantly associated with LN metastasis (OR, 1.01; 95% CI: 0.99, 1.03; *P* = 0.35) (Table 4).

**Table 4:** Results of the multivariate analysis of the pathological and morphological model and dual-energy computed tomography parameters with the shortest diameter of the lymph node model

Characteristics	Odds ratio	95% CI	*P* value
Pathological and morphological model			
Estrogen receptor status	0.78	0.18, 3.44	0.74
Ki67 index	1.01	0.99, 1.03	0.35
Hilum of LN	0.21	0.06, 0.72	0.014
Shortest diameter of LN	2.49	1.65, 3.77	< 0.001
DECT parameters with the shortest diameter of the LN model			
Shortest diameter of LN	2.22	1.47, 3.35	< 0.001
Attenuation at 40 keV	1.01	0.99, 1.03	0.35
The ROD of attenuation at 40 keV	2.00	1.13, 3.53	0.017

*CI* confidence interval, *LN* lymph node, *ROD* rate of difference

### Diagnostic ability of simple DECT parameters and the similarity of DECT parameters for LN metastasis

**Fig. 3:**
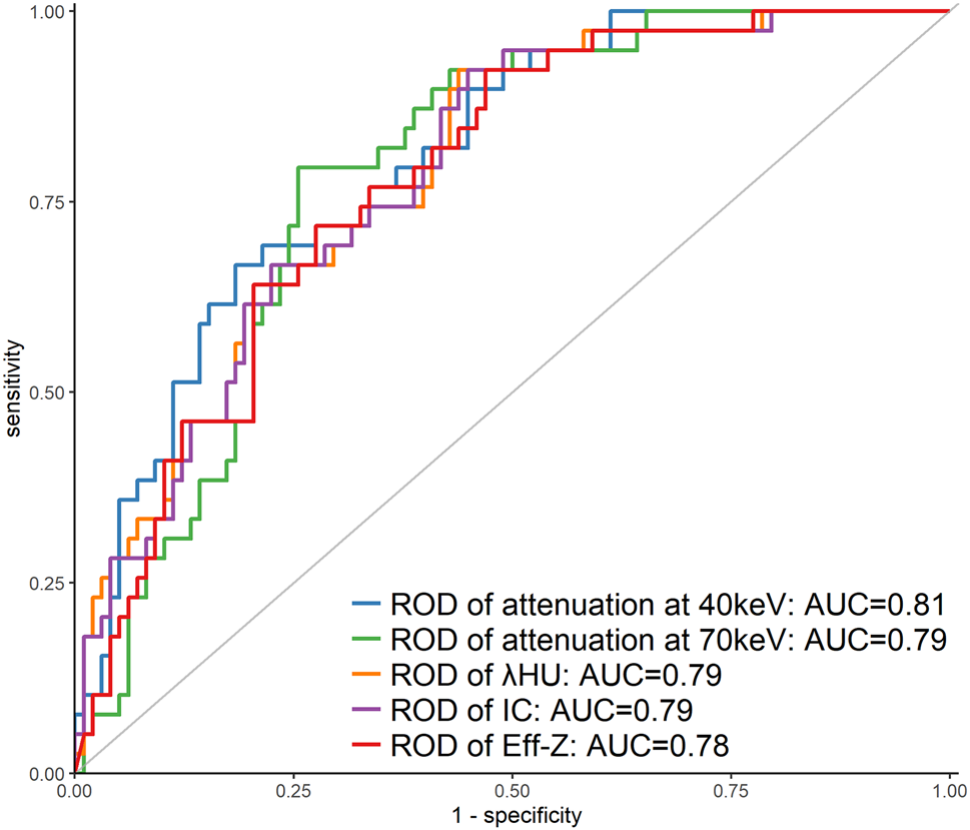
Receiver operating characteristic curves of the rate of difference (ROD) of quantitative dual-energy computed tomography parameters between the primary lesion and axillary lymph node in all patients. *ROD* rate of difference, *λHU* slope of the spectral Hounsfield unit curve, *IC* iodine concentration, *Eff-Z* effective atomic number, *AUC* area under the curve

**Fig. 4:**
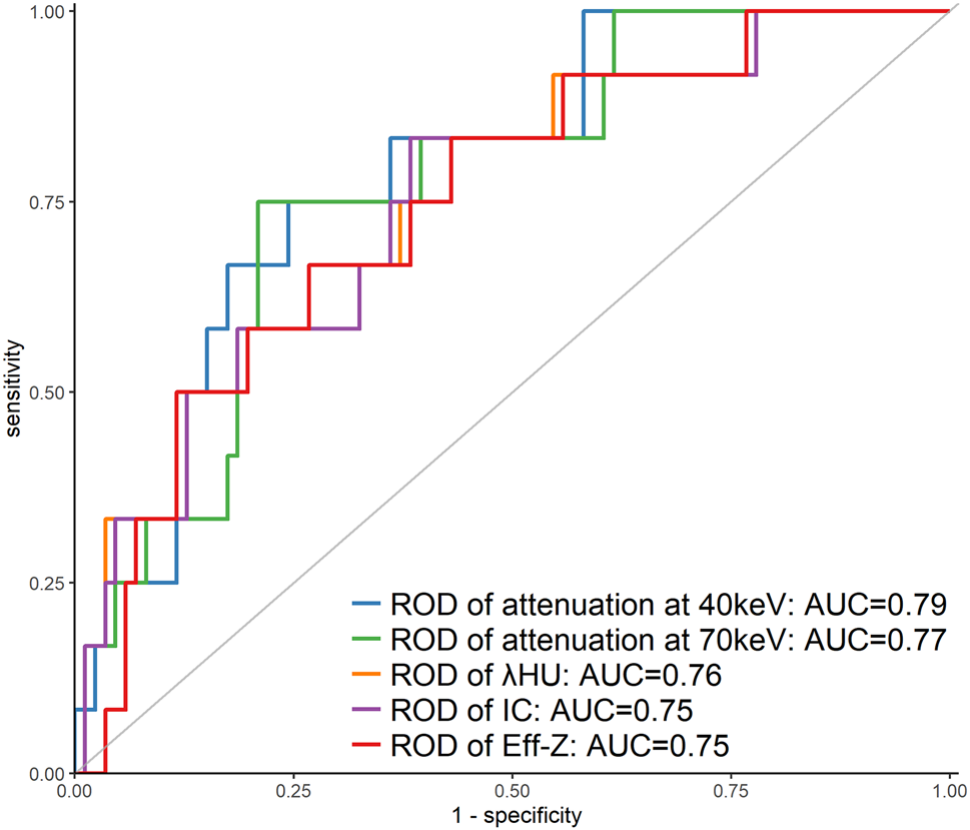
Graphs showing receiver operating characteristic curves of the rate of difference (ROD) of quantitative dual-energy computed tomography parameters between the primary lesion and axillary lymph node in patients with a lymph node diameter < 5 mm. *ROD* rate of difference, *λHU* slope of the spectral Hounsfield unit curve, *IC* iodine concentration, *Eff-Z* effective atomic number, *AUC* area under the curve

As shown in Figs. 3 and 4 and Table 5, the ROC curve derived from the ROD between the primary lesion and axillary LN showed that 0.032–0.232 (attenuation at 40 keV and 70 keV, λHU, IC, and Eff-Z) was the optimal threshold to distinguish LN metastasis (sensitivity 66.7–92.3%, specificity 56.1–81.6%, AUC 0.78–0.81). The AUC of the ROD between the primary lesion and axillary LN in the delayed phase was slightly higher than that of the simple delayed-phase DECT parameters. Although the shortest diameter of the LN was limited to < 5 mm (LN metastasis group: *n* = 12, LN non-metastasis group: *n* = 86), similar results were obtained (sensitivity 57.0–83.8%, specificity 61.6–83.3%, AUC 0.73–0.78, threshold 0.03–0.20).

**Table 5:** Results of the receiver operating characteristic curve analysis of the quantitative dual-energy computed tomography parameters and rate of difference of those parameters between the primary lesion and axillary lymph node in the delayed phase for predicting metastatic axillary LN in the total cohort and cohort with a lymph node size < 5 mm

Parameters	AUC	Threshold	Sensitivity (%)	Specificity (%)
Full cohort, LN metastasis group (*n* = 39), LN on-metastasis group (*n* = 98)				
Simple DECT parameters				
Attenuation at 40 keV	0.76	227.6	71.8	72.4
Attenuation at 70 keV	0.78	96.3	64.1	83.7
λHU	0.73	4.3	79.5	61.2
IC	0.73	22.8	79.5	61.2
Eff-Z	0.72	8.9	79.5	61.2
The ROD of DECT parameters				
Attenuation at 40 keV	0.81	0.105	66.7	81.6
Attenuation at 70 keV	0.79	0.127	79.5	74.5
λHU	0.79	0.231	92.3	56.1
IC	0.79	0.232	92.3	56.1
Eff-Z	0.78	0.032	92.3	56.1
LN (shortest diameter < 5 mm), LN metastasis group (*n* = 12), LN on-metastasis group (*n* = 86)				
Simple DECT parameters				
Attenuation at 40 keV	0.74	198.2	91.7	53.5
Attenuation at 70 keV	0.75	95.1	83.7	91.7
λHU	0.70	3.90	91.7	47.7
IC	0.70	20.7	91.7	47.7
Eff-Z	0.69	8.81	93.3	45.2
The ROD of DECT parameters				
Attenuation at 40 keV	0.79	0.133	75.0	75.6
Attenuation at 70 keV	0.77	0.120	75.0	79.1
λHU	0.76	0.204	83.8	61.6
IC	0.75	0.204	83.8	61.6
Eff-Z	0.75	0.030	57.0	83.3

*AUC* area under the curve, *DECT* dual-energy computed tomography, *Eff-Z* effective atomic number, *IC* iodine concentration, *LN* lymph node, *λHU* slope of the spectral Hounsfield unit curve, *ROD* rate of difference

## Discussion

In this study, the quantitative DECT parameters, including the λHU, IC, and attenuation values at 40 keV and 70 keV, were useful for predicting LN metastasis, as previously reported [18]. However, these DECT parameters may be influenced by differences in the CT scanner, scanning protocols, and injection protocols of the contrast medium. This problem limits the widespread use of this method in clinical practice. Herein, we revealed that the similarity of DECT parameters between the primary breast cancer lesion and axillary LN was also useful for predicting LN metastasis. Multivariable analysis showed that the ROD, which reflects the similarity between the primary lesion and LN, of delayed-phase DECT parameters (attenuation at 40 keV), was significantly associated with LN metastasis more than delayed-phase simple DECT parameters. Moreover, the AUC for predicting LN metastasis using the ROD of DECT parameters was higher than that of simple DECT quantitative data. Assessing the similarity between the primary lesion and LN metastasis could be a CT scanning factor-independent assessment method. Therefore, the evaluation of similarity seems to be superior to the evaluation of simple quantitative DECT parameters.

Multivariate analysis showed that the shortest diameter of the LN was most associated with LN metastasis. This finding was different from that in another study, in which the venous-phase λHU was more significant than the morphologic parameters [18]. Differences in cohort and imaging conditions may have contributed to these differences, but the details are unknown.

US or MRI is useful conventionally to detect axillary LN metastasis of breast cancer [23–25]. LN metastasis was considered present when the LN size was > 5 mm in diameter. [26–28]. However, the LN size only had poor sensitivity, and small metastatic LNs were also present [24]. In our cohort, 12 patients (30.8%) had small metastatic LNs that were < 5 mm in size. The ROD of delayed-phase DECT parameters among primary cancer and the axillary LN had AUC values of 0.73–0.78, even in cohorts with small LNs (shortest diameter < 5 mm). Therefore, these parameters (RODs of the DECT parameters) are one of the useful tools for predicting LN metastasis in breast cancer.

In metastatic LNs, tumor cells migrate via the afferent lymphatic vessels into the subcapsular sinus, and the growing metastasis replaces the LN tissue [29]. Although these are different tissues, metastatic LNs are expected to become similar to primary lesions in hemodynamics or image features. Our study found a moderate correlation of DECT parameters in the delayed phase between the primary lesion and axillary LN with metastasis. The ROD between the DECT parameters of the primary lesion and that of the axillary LN was significantly smaller in the LN metastasis group than in the LN non-metastasis group. The similarity between the primary lesion and metastatic LN can be used regardless of the CT manufacturer or imaging protocol. This similarity may be used as a simple method for predicting LN metastasis and can be used for other tumors. However, the examination of this similarity of DECT parameters requires further study.

Our study has some limitations. First, this was a single-institution retrospective study with a small number of patients. Therefore, the data distribution was unbalanced. Second, axillary LNs without metastasis were too small to place the ROI, so some cases were excluded. Third, cases of N1mi (*n* = 3) were excluded from this study, potentially causing selection bias. However, the number of patients with N1mi was small, so the impact is limited. Fourth, we only investigated one axillary LN per patient, because it is difficult to link all excised LNs on CT images. Additionally, in the SLN positive cases, we were almost certain the LNs on DECT matched LNs pathologically diagnosed as metastasis, but not absolutely. Finally, the similarity of DECT parameters between the primary lesion and metastatic LN has little evidence to date. The similarity between the tissues of LN metastasis and primary breast cancer is not unclear. Further studies are required.

In conclusion, the similarity of the delayed-phase DECT parameters could be a more useful tool for predicting LN metastasis than assessing the simple DECT parameters in patients with breast cancer, regardless of the size of the LNs.
